# Clinical effect of nice knot-assisted minimally invasive titanium elastic nail fixation to treat Robinson 2B midshaft clavicular fracture

**DOI:** 10.1186/s12891-024-07197-4

**Published:** 2024-01-13

**Authors:** Yongqiang Kang, Qingqing Zhang, Yunhong Ma, Ming Zhou, Xueyuan Jia, Fang Lin, Yongwei Wu, Yongjun Rui

**Affiliations:** 1 Medical College, Soochow University, Suzhou, Jiangsu China; 2 Department of Traumatic Orthopedics, Wuxi Ninth People’s Hospital, Soochow University, Liangxi Road, No.999, Binhu District, Wuxi, Jiangsu China

**Keywords:** Clavicular fracture, Intramedullary fixation, Nice knot, Displaced midshaft, Minimally invasive

## Abstract

**Background:**

The treatment of completely displaced midshaft clavicle fractures is still controversial, especially Robinson 2B fractures. Titanium elastic nail (TEN) fixation is a good option for simple fractures, but no reports exist on its use in complex fractures. This study aimed to present a surgical method using the Nice knot-assisted TEN fixation to treat Robinson 2B midshaft clavicular fractures.

**Methods:**

A retrospective analysis of 29 patients who underwent fixation with TEN and had a 1-year postoperative follow-up between 2016 and 2020 was performed. The fractures were classified as Robinson type 2B1 in 17 cases and type 2B2 in 12 cases. Length of the incision, postoperative shoulder function Disability of Arm Shoulder and Hand (DASH) score and Constant score, complications rate, and second surgical incision length were recorded.

**Results:**

The length of the incision was 2–6 cm (average 3.7 cm). All incisions healed by first intention, and no infection or nerve injury occurred. The Constant score was 92–100 (average 96) and the DASH score was 0-6.2 (mean, 2.64). TEN bending and hypertrophic nonunion occurred in one case (3.4%) and implant irritation occurred in four cases (13.8%) Fixation implants were removed at 12–26 months (mean, 14.6 months) after surgery, and the length of the second incision was 1-2.5 cm (average 1.3 cm).

**Conclusions:**

Intramedullary fixation by TEN is approved as a suitable surgical technique in clavicular fracture treatment. Nice knot-assisted fixation provides multifragmentary fracture stabilization, contributing to good fracture healing. Surgeons should consider this technique in treating Robinson 2B midshaft clavicular fractures.

**Trial registration:**

Retrospectively registered. This study was approved by the Ethics Committee of Wuxi Ninth People’s Hospital (LW20220021).

## Background

Clavicle fractures predominantly occur in the middle segment of the collar bone, accounting for 76–81% of all clavicle fractures. Recent studies have suggested that surgical treatment via plate fixation results in a higher union rate and better subjective outcome scores than nonsurgical treatment [1–3]. Plate fixation is considered the gold standard treatment for midshaft clavicular fractures [4]. However, this treatment is associated with soft tissue damage, extensive dissection, excessive intraoperative bleeding, and the risk of peripheral nerve and blood vessel damage. Additionally, significant epithelial nerve damage can occur during plate removal surgery.

Titanium elastic nail (TEN) fixation has been associated with a high rate of union [5]. However, TEN fixation is used for simple midshaft fractures [6–8], with no published study about TEN fixation of a Robinson 2B midshaft clavicular fracture.

In this study, the Nice knot formed by high-strength polyblend sutures was used to fix and stabilize multifragmentary clavicle midshaft fractures, and the TEN was inserted through the medial entry point. The aim of this study was to present a surgical method–Nice knot-assisted TEN fixation–for treating Robinson 2B midshaft clavicular fractures.

## Methods

### Patients

The inclusion criteria were (1) midshaft clavicular fractures; (2) 18–65 years old; (3) newly closed fracture (within 2 weeks after injury); (4) > 12 months follow-up; (5) undergoing the second surgery in our hospital. Patients with open or pathological fractures and those with vascular nerve injury in the ipsilateral limb and any other injury that affects limb function were excluded.

From January 2016 to April 2020, 29 patients with Robinson 2B midshaft clavicular fractures were treated using Nice knot-assisted minimally invasive TEN fixation at the same hospital. Of these, 14 patients were male, and 15 patients were female, and their ages ranged from 17 to 63 years. Of the 29 patients, 17 sustained “simple” displaced two-part midshaft clavicle fractures (Robinson 2B.1), and the remaining 12 patients sustained displaced “complex multifragmentary” clavicle fractures, comprising three-part and four-part fractures (Robinson 2B.2). All patients underwent intramedullary fixation using 2.0 to 3.0 mm TENs.

This study was approved by the Ethics Committee of Wuxi Ninth People’s Hospital (LW20220021), and the protocol adhered to the Helsinki Declaration of 1975, as revised in 2008. Informed consent was obtained from all patients before their inclusion in the study.

### Operative technique

Preoperative planning included obtaining anteroposterior radiographs, CT images, and three-dimensional (3D) reconstruction images of the clavicle to determine the degree of bone displacement and evaluate intermediary fragments. Next, the diameter of the medullary canal at the narrowest part of the middle segment of the clavicle was measured, and the diameter of the TENs was evaluated. Larger intramedullary nails were preferred.

**Fig. 1:**
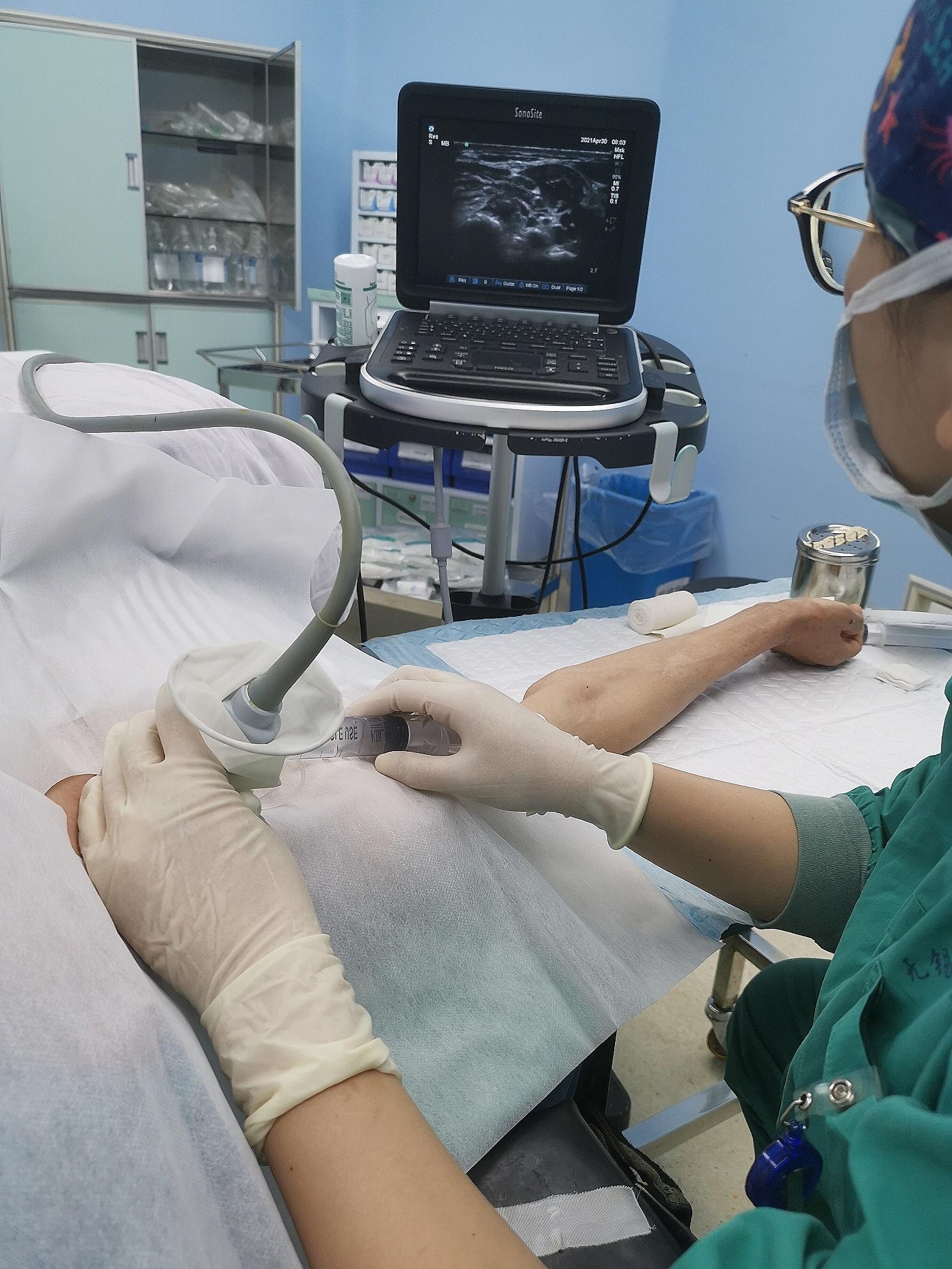
A cervical plexus block was administered under ultrasound guidance

**Fig. 2:**
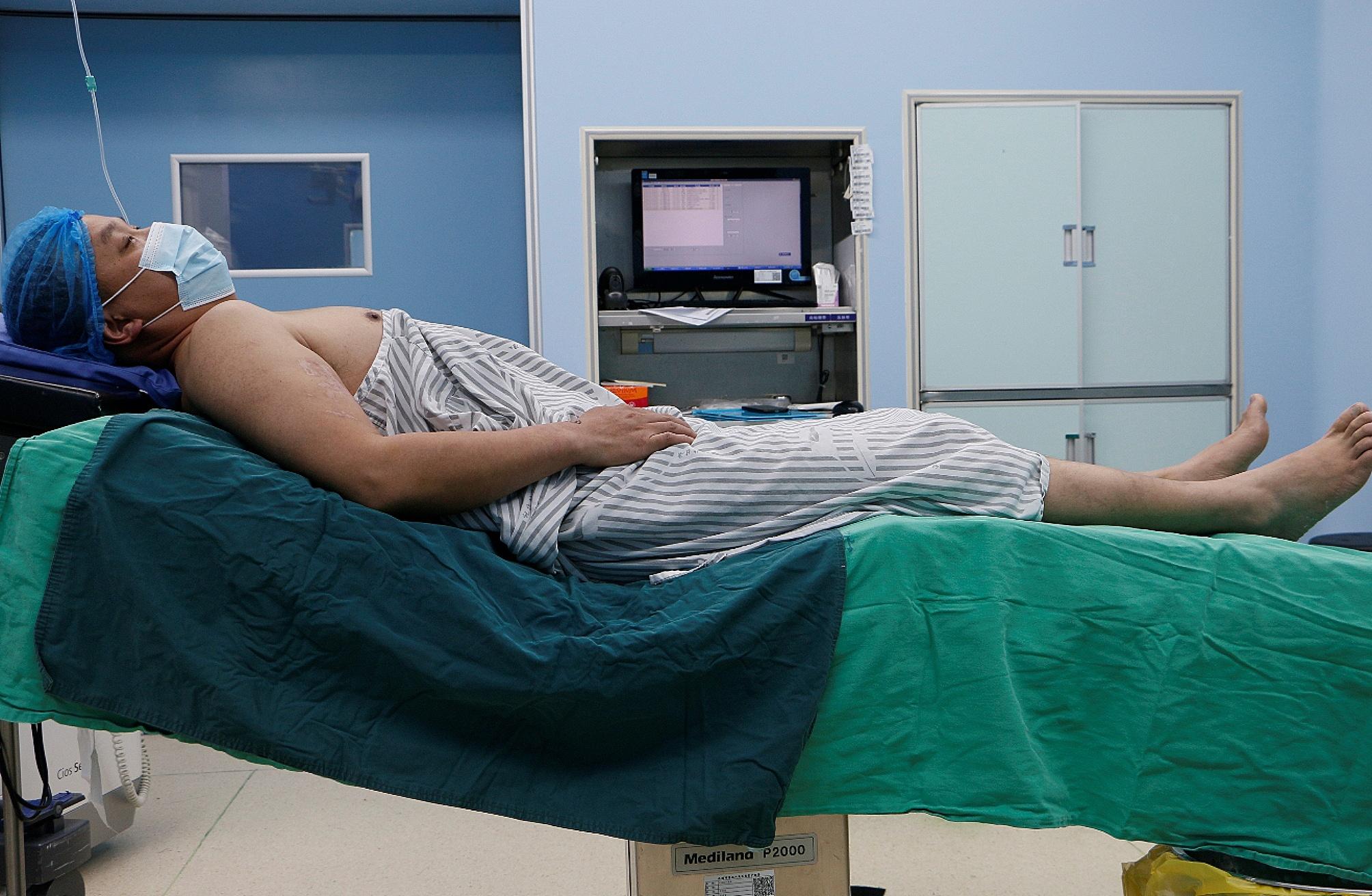
The patient was placed in the beach chair position

**Fig. 3:**
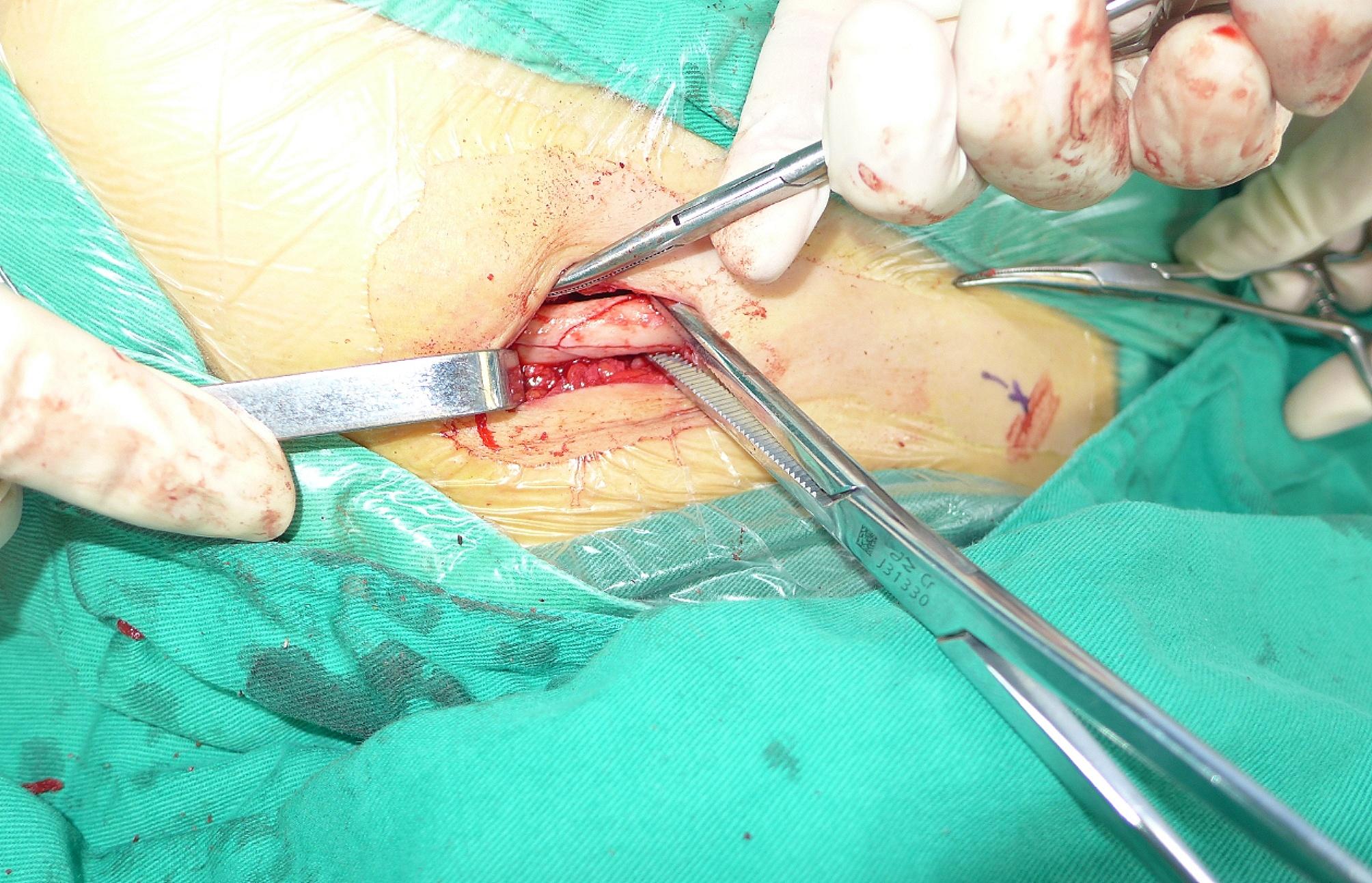
An incision was made as appropriate. The fracture was reduced after cleaning, and temporary fixation was achieved by using Kocher forceps

After the preoperative preparation, surgery was performed. All surgeries were conducted by the same surgeon. First, a cervical plexus block was administered under ultrasound guidance (Fig. 1). The patient was placed on a radiolucent table in the beach chair position with the C-arm positioned cranially or laterally at the injured side (Fig. 2). A 1–3 cm skin incision was made in the plane of the fracture directly to the bone. If reducing the fracture was difficult, the incision was extended appropriately. The fracture was reduced after cleaning, and temporary fixation was achieved using Kocher forceps (Fig. 3). If the medullary canal at the fracture site was very narrow, the medullary canal was opened by a 2.5-mm Kirschner wire.

**Fig. 4:**
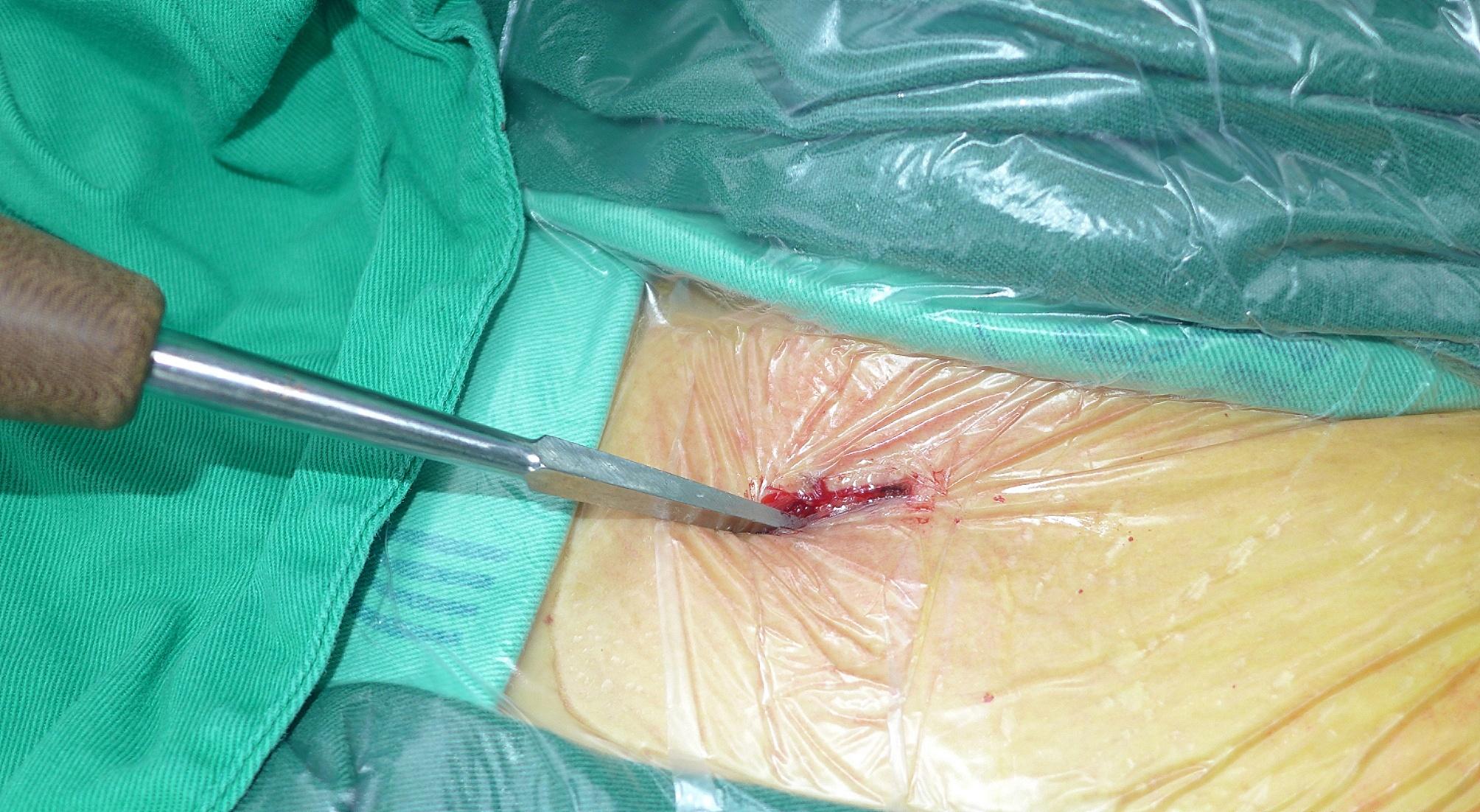
An awl was used to widen the oblique opening hole in line with the clavicle axis, to facilitate nail entry

**Fig. 5:**
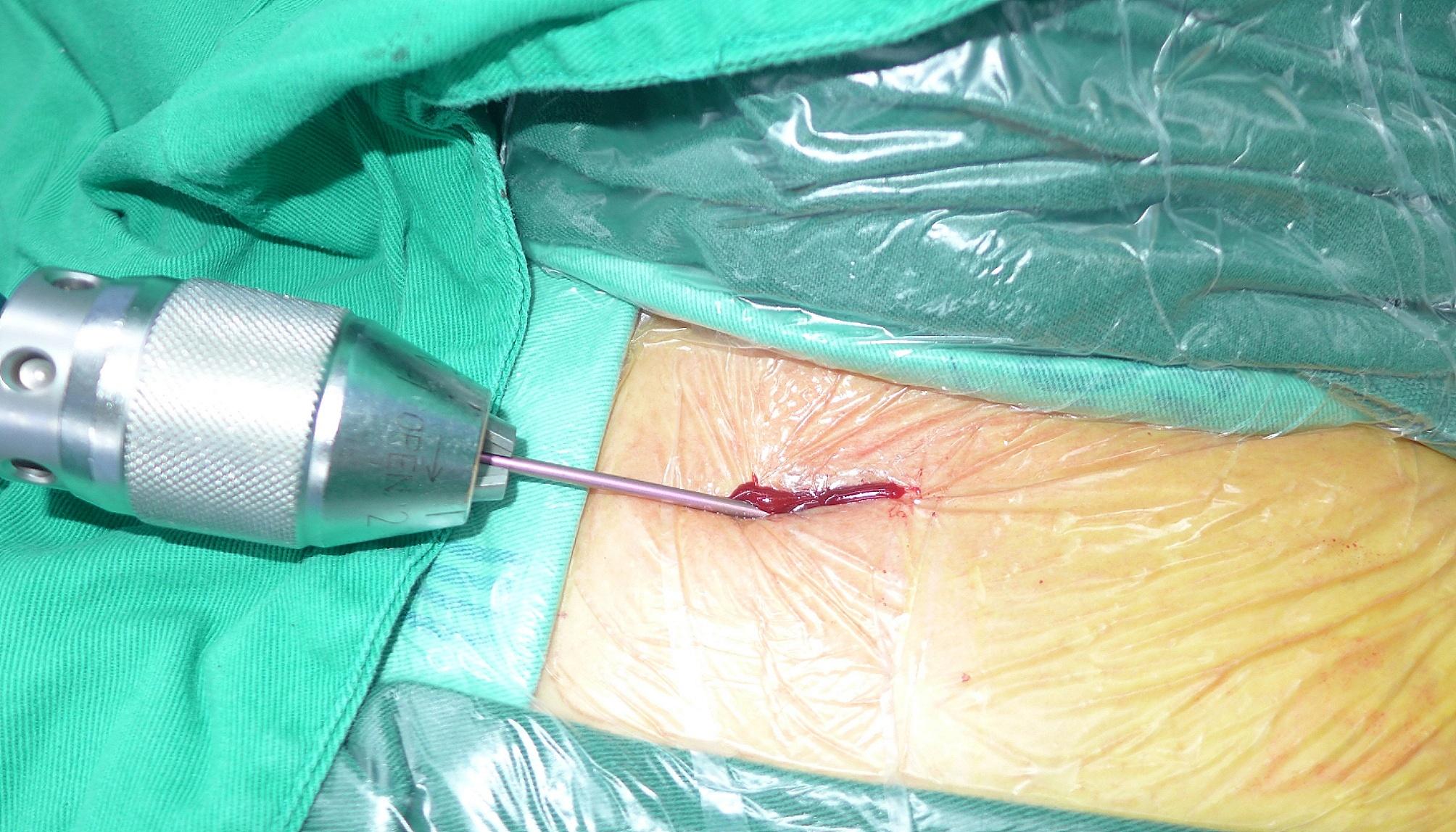
The nail was fixed and inserted into the medullary canal

**Fig. 6:**
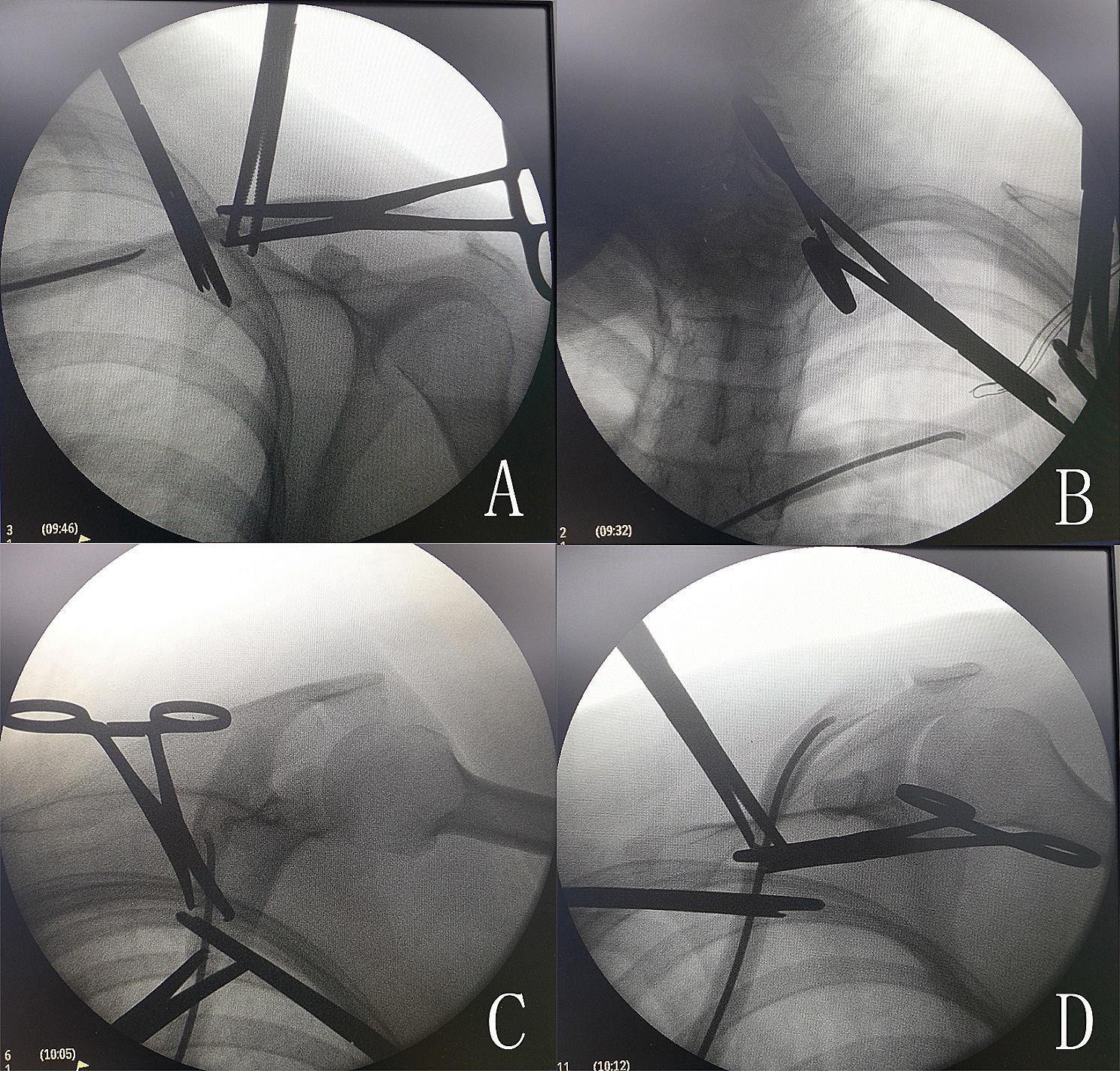
The direction of the elastic nail was continuously adjusted under C-arm fluoroscopy to ensure that it had reached the lateral side of the clavicle through the fracture site

Next, a 1 cm skin incision was made 1–2 cm lateral to the sternoclavicular joint directly to the bone. The medullary canal was also opened by a 2.5-mm Kirschner wire. An awl was used to widen the oblique opening hole in line with the clavicle axis to facilitate nail entry (Fig. 4). The nail was fixed and inserted into the medullary canal (Fig. 5). The direction of the elastic nail was continuously adjusted under C-arm fluoroscopy to ensure that the nail reached the lateral side of the clavicle through the fracture site (Fig. 6A-D).

**Fig. 7:**
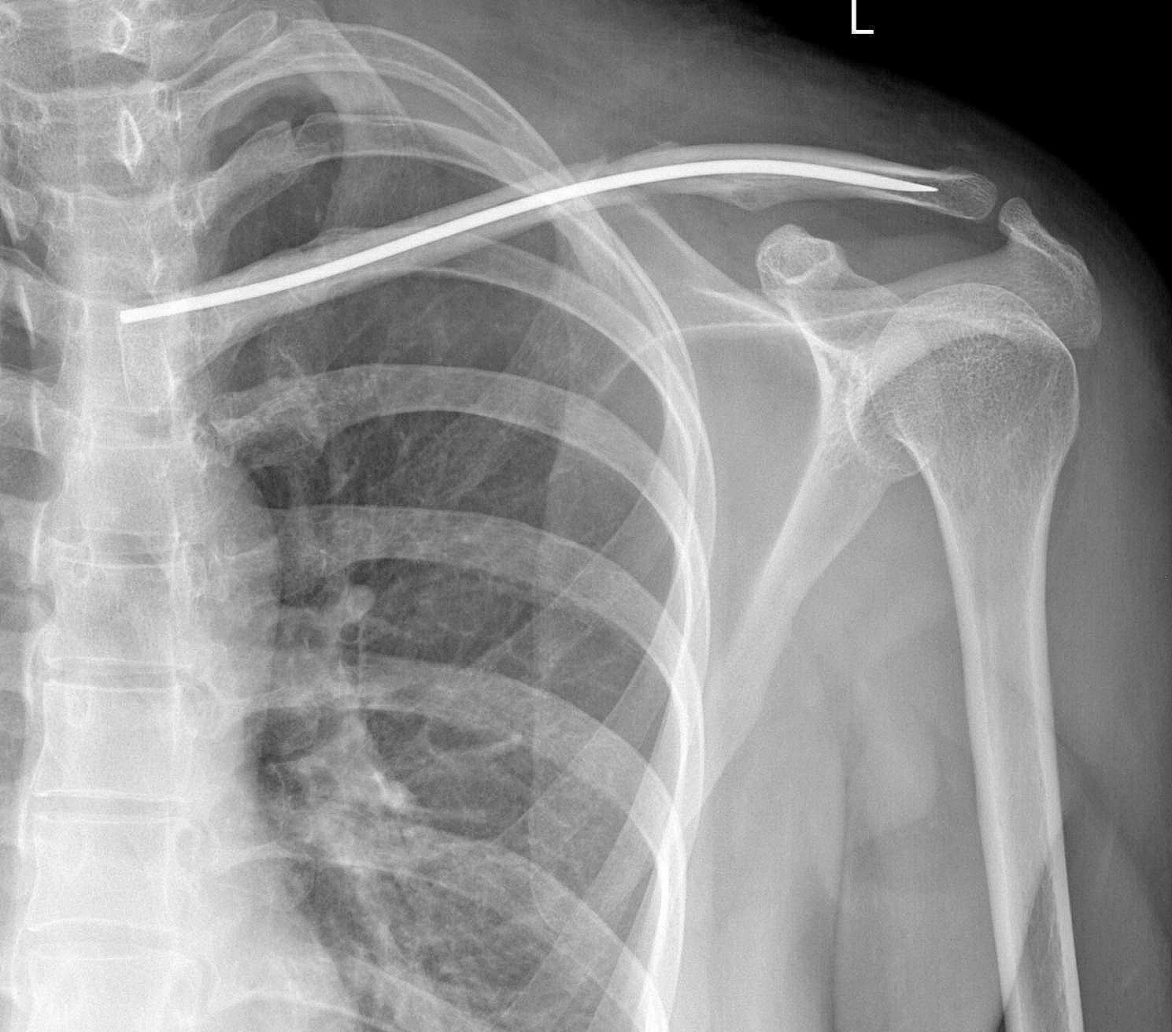
The tip of the nail was aligned with the flat medullary canal and positioned anteriorly as the lateral curvature was reached

**Fig. 8:**
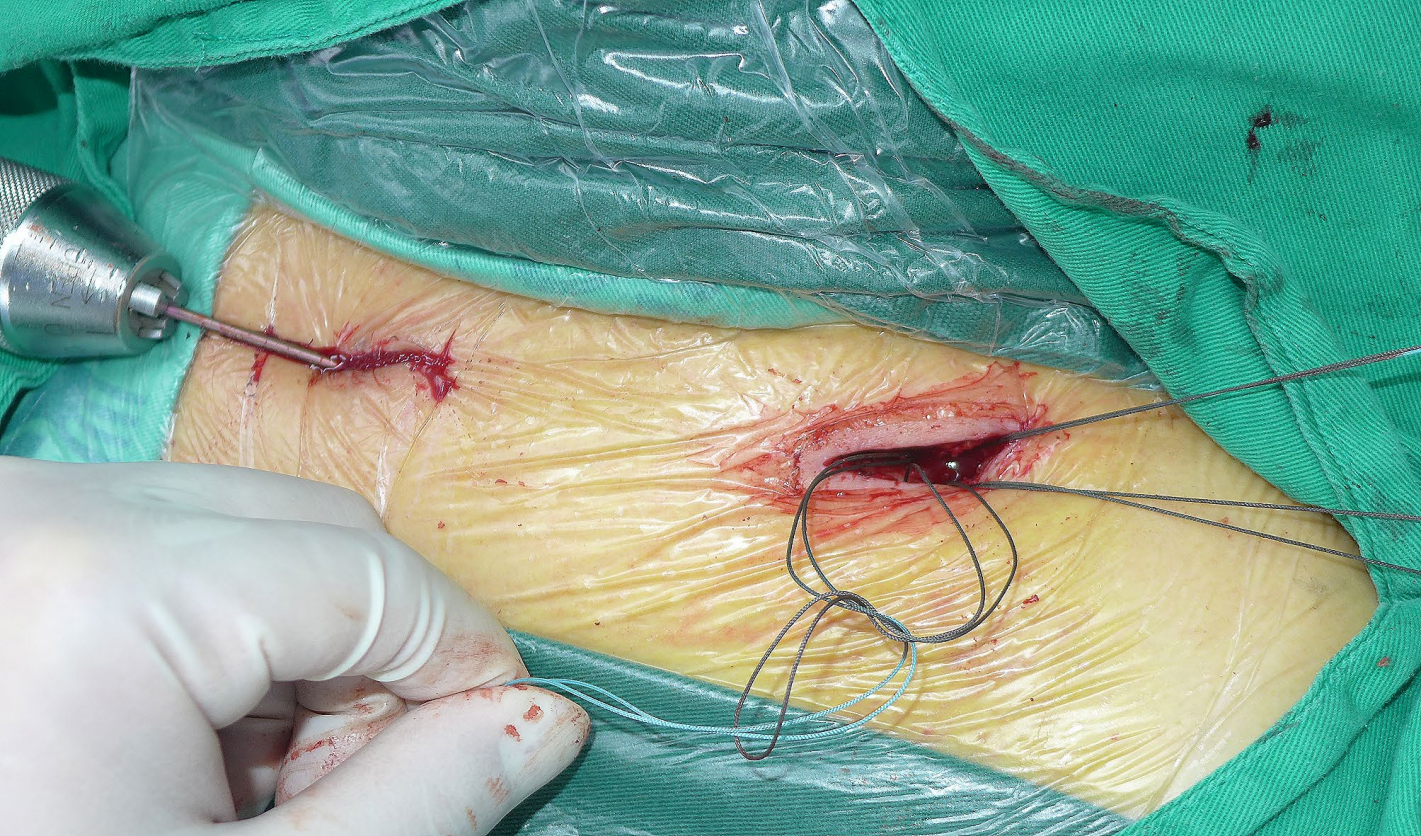
A no. 2 Ethibond non-absorbable thread (Johnson & Johnson, United States) was used to fix the fracture with the Nice knot

After inserting the nail into the lateral side, attention was paid to the flattened shape and anterior curvature of the lateral end of the clavicle. The tip of the nail was aligned with the flat medullary canal and positioned anteriorly as the lateral curvature was reached (Fig. 7). The Kocher forceps was then removed from the fracture site, and no. 2 Ethibond non-absorbable thread (Johnson & Johnson, United States) was used to fix the fracture with the Nice knot (Fig. 8). Lastly, the nail was cut down to the bone with a wire cutter, and 5 mL of tranexamic acid was administered in the incision site to reduce postoperative bleeding.

### Postoperative physical recovery

Each patient wore a simple sling for 4 weeks for comfort but was encouraged to take the arm out and move the wrist and elbow. Passive function training of the shoulder joint began on the second postoperative day, but active shoulder motion was prohibited until the 4th to 6th week, with shoulder joint exercises in all directions starting at 6 weeks to 3 months after surgery. Active motion of the elbow, wrist, and hand were not restricted. Resistance training was performed at the last phase of physical recovery.

### Curative effect evaluation index

The total lengths of the surgical incisions at the fracture site and sternoclavicular joint were recorded. Radiography was performed monthly after surgery until the fracture had healed. One year after surgery, the Constant score and the disability of arm, shoulder, and hand (DASH) score were used to evaluate each patient’s shoulder joint function. The occurrence of complications, including incision site infection, skin irritation, fracture nonunion, symptoms of nerve injury, and failure of internal fixation, were recorded. The length of the incision for the second surgery to remove the fixation implant was also recorded.

## Results

Lengths of incisions ranged from 2 to 6 cm (average 3.7 cm). All incisions healed by first intention, and no infection or nerve injury occurred. The Constant score ranged from 92 to 100 (average 96), and the DASH score ranged from 0 to 6.2 (average 2.64). TEN bending and hypertrophic nonunion occurred in one case (3.4%), and implant irritation occurred in four cases (13.8%) after surgery. The patient with bending and hypertrophic nonunion became active shortly after the surgery, and the elastic nail was bent as appropriate at 3 months after surgery. After the subsequent immobilization, no fracture healing was observed. At 9 months after surgery, the patient was re-hospitalized due to hypertrophic nonunion, and the elastic nail was removed. New internal plate fixation and bone grafting were performed. The fracture healed after 4 months.

Four patients developed local protrusion of the tail of the elastic nail after surgery, irritating the skin. The tail of the nail was cut off under local anesthesia, and the tail stump was rotated and buried in the soft tissue above the clavicle at the sternoclavicular joint. The fracture healed well in these patients and the skin irritation did not reappear during the period.

**Fig. 9:**
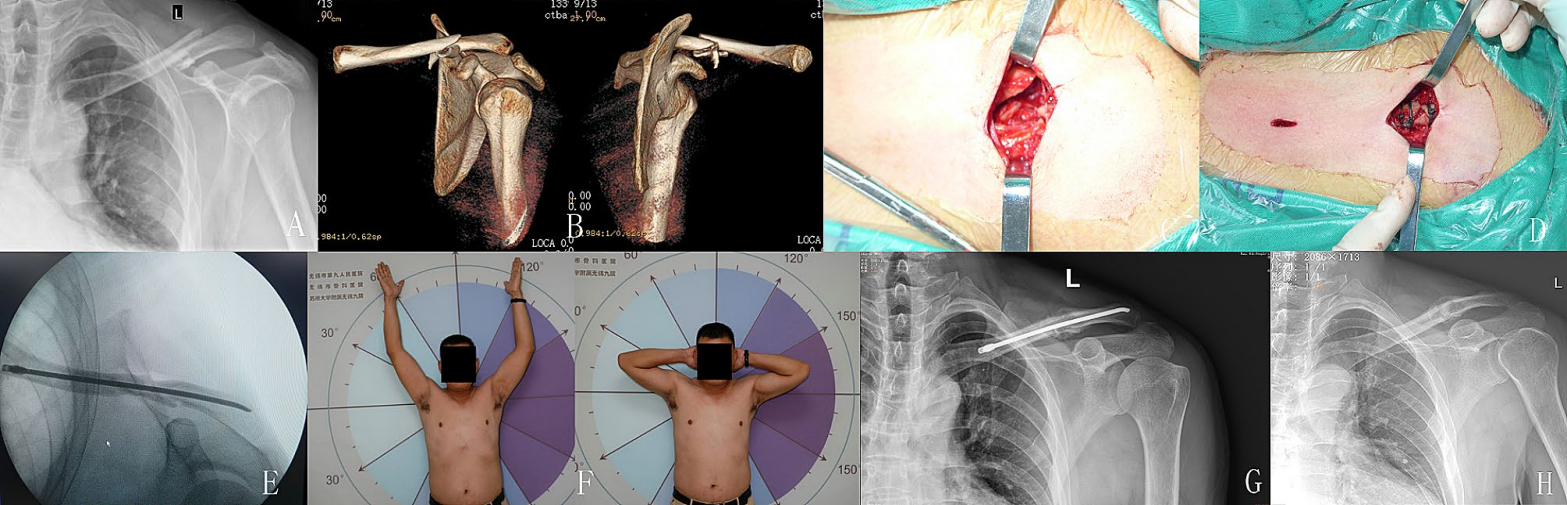
A 41-year-old man had a traffic injury, resulting in a Robinson 2B2 left midshaft clavicle fracture. **A/B**: Radiography and CT of the fracture. **C**: Intraoperative fracture displacement. **D**: The fracture was reduced, and no. 2 Ethibond non-absorbable thread was used to fix the fracture with the Nice knot. **E**: Follow-up radiography of the fracture after surgery. **F**: Clinical appearance at 8 months after surgery. **G/H**: Radiography confirms fracture healing at 8 months after surgery, and the implant was removed

Implant removal was performed at 12–26 months (average 14.6 months) after surgery with the length of incision ranging from 1 to 2.5 cm (average 1.3 cm) (Tables 1 and 2). A typical case is shown in Fig. 9.

**Table 1:** Clinical data of patients with Robinson 2B.1 fractures

Patient	Age(yr)/Sex	Cause of injury	Side	Length of incision (cm)	Constant score	DASH score	Complications
1	52/F	fall	right	4	98	0	none
2	20/M	fall	left	6	98	2.6	ESIN bending, hypertrophic nonunion
3	50/F	traffic accident	left	6	86	10	none
4	17/M	traffic accident	right	2	100	2.1	none
5	44/M	traffic accident	left	3	98	4	none
6	15/F	fall	right	3	95	3.6	none
7	56/M	traffic accident	left	5	96	4	none
8	55/M	fall	left	2.5	96	3	none
9	49/F	traffic accident	right	3.5	98	1.3	implant irritation
10	42/F	fall	left	2	100	1.6	none
11	18/F	fall	right	2.5	98	3.2	none
12	49/F	traffic accident	right	3.5	98	3.4	none
13	39/F	traffic accident	left	5	95	2.6	none
14	21/F	traffic accident	left	2.5	96	1.2	none
15	46/F	traffic accident	right	2	98	1.4	none
16	50/M	fall	left	3.5	99	3.2	none
17	63/F	traffic accident	right	3.5	88	2.4	implant irritation

**Table 2:** Clinical data of patients with Robinson 2B.2 fractures

Patient	Age(yr)/Sex	Cause of injury	Side	length of incision (cm)	Constant score	DASH score	Complications
1	41/F	traffic accident	left	5	98	1.2	affected sleep
2	52/F	fall	right	5.5	92	4.2	none
3	55/M	traffic accident	left	5.5	98	3.6	implant irritation
4	59/M	crushing accident	left	3	96	6.2	none
5	63/F	fall	left	3.5	95	3.1	none
6	54/M	fall	left	2.5	100	1.2	none
7	39/M	traffic accident	left	5	100	0	none
8	27/F	traffic accident	left	4	99	1.6	none
9	55/M	traffic accident	left	4.5	92	0	none
10	62/M	fall	right	3.5	89	1.6	none
11	47/M	fall	right	3.5	96	2.2	none
12	47/M	traffic accident	left	4	92	1.9	implant irritation

## Discussion

The three main treatment methods for Robinson 2B middle clavicle fractures in adults are open reduction and internal plate fixation, internal fixation using elastic nails, and external fixation [9]. Internal plate fixation is considered the gold standard for middle clavicle fractures, and it has obvious advantages [10]. However, studies have reported a nonunion or malunion rate of up to 15.1%. Patient dissatisfaction related to pain was up to 31%, and neurological complications, cosmetic problems, limb shortening, and malunion can necessitate subsequent surgeries [11–13]. With improving living standards, patients increasingly require good aesthetical outcomes of postoperative scarsand appreciable final clavicle function, particularly female patients.

In 2003, Jubel et al. [14] first described TEN fixation in a cohort study. One-third of the cohort had sustained multifragmentary fractures. Gareth et al. reported that intramedullary fixation of displaced midshaft clavicle fractures (Robinson 2b.1) using plates had an equivalent nonunion rate, and similarly low complication and reoperation rates [15]. There were also smaller scars, less soft tissue disruption, and less soft tissue irritation by the implant in intramedullary fixation. Some other studies that evaluated TEN fixation reported high union rates (96–100%) [5, 16, 17]. However, these studies reported the treatment of simple fractures of the middle clavicle. Therefore, we could consider the intramedullary nail technology a satisfactory method for treating simple middle clavicle fractures. However, regarding complex multifragmentary clavicle fractures, the current intramedullary nail technology has not been well applied due to the instability of comminuted bone fixation.

Bakota et al. [1] reported a comparative study with a modified Murray and Schwarz 2.5-mm Kirschner wire intramedullary technique in the fixation of displaced Robinson 2B.1 and Robinson 2B.2 fractures midshaft clavicle fractures. The study concluded that intramedullary clavicle fixation using a 2.5-mm Kirschner wire was a safe surgical technique. Further, Robinson 2B.1 fractures treated by 2.5-mm Kirschner wire fixation had relatively improved outcomes compared to displaced Robinson 2B.2 fractures in terms of nonunion and reoperation rates. Autohors noted that an ideal treatment method for Robinson 2B.2 middle clavicle fractures by intramedullary nailing was still being explored.

Suture binding technique is used to increase the stability of the fracture block; improving intramedullary nailing treatment of the complex multifragmentary clavicle fractures. The Nice knot was invented by Pascal Boileau [18]. It is a high-tension knot with strong tension resistance and can play a role in fixing the fracture after reduction; this type of knot provides gradual tightening. In the present study, several double-stranded Nice knots were set in multiple directions and tightened progressively. This approach is similar to one described by Boileau et al. where Nice knots were used to fix greater tuberosity humeral fractures and favorable clinical outcomes were observed [19]. Chen et al. used the Nice knot as an auxiliary reduction technique in displaced comminuted patellar fractures and found the technique satisfactory and comparable to traditional reduction techniques [20]. No study has reported Nice knots for midshaft clavicle fracture treatment.

In our study, Nice knots were combined with TEN fixation to avoid problems accompanying steel plate fixation and we have found notable advantages. First, only small incisions were made above the fracture site and sternoclavicular joint, and there was no need to strip the periosteum extensively. The fracture could be anatomically reduced with little soft-tissue damage. The surgical incisions were only 2–6 cm long, significantly shorter than traditional incisions (8.3 ± 1.6 cm) [4], and intraoperative bleeding was minimal. The elastic nail penetrated the medullary cavity from the proximal clavicle, avoiding damage to the supraclavicular nerve. The second operation included the removal of the intramedullary fixation implant only through the original incision exposed at the proximal clavicle site. No patient had symptoms of nerve injury after surgery, and the length of the surgical incision in the second surgery was only 1–2.5 cm. The Nice knot was used to stabilize the fracture, including its free wedge fragment, ensuring safer early postoperative functional training and increasing the stability of intramedullary nail fixation. All patients in this group began shoulder joint passive motion training at the second day after surgery. Both PCS and MCS were significantly higher. Considering the results of Robinson et al. [21] who reported a DASH score of 3.4 and a Constant score of 92.0after plate fixation, average DASH score 2.64 and average Constant score 96 from our study could be taken as satisfactory. Except for one patient who began active motions too early and caused nonunion, none of the other 28 patients experienced complications, including failure of internal fixation. Fixation implant could not be touched under the skin, thus the implant related discomfort was avoided. The cost of intramedullary nails is significantly lower than that of steel plates, reducing the medical expenses of the treatment.

The use of Nice knot-assisted minimally invasive TEN fixation has a high rate of bone union and soft-tissue complications. In our study, four cases included medial skin irritations requiring surgical shortening of the nail (13.8%). Van der Meijden et al. [17] reported 16.1% of skin irritations in intramedullary fixation, a result similar to our study. The problem of skin and local soft-tissues irritation should be investigated further.

## Conclusions

We have demonstrated the safety and efficacy of the Nice knot-assisted minimally invasive TEN fixation in treating Robinson 2B midshaft clavicular fractures. In terms of incision length, clinical appearance, avoidance of nerve damage and second operation time and incision length, this procedure has more advantages than open reduction and locking plate internal fixation. It also overcomes the problem of poor rotation resistance with a high bone union rate. The procedure provides more options for the clinical treatment of middle clavicle fractures. However, further studies with a larger sample size and longer follow-up time should be performed for more relevant clinical efficacy assessments.

## Data Availability

All data generated or analyzed during this study are included in this published article.

## Abbreviations

DASH: The disability of arm, shoulder, and hand
TEN: Titanium elastic nail
